# Early short-term abdominal paracentesis drainage in moderately severe and severe acute pancreatitis with pelvic ascites

**DOI:** 10.1186/s12893-023-02269-z

**Published:** 2023-11-27

**Authors:** Jie Huang, Lei Li, Ying Chen, Enqiang Mao, Hongping Qu

**Affiliations:** 1 Department of Critical Care Medicine, Ruijin Hospital, Shanghai Jiao Tong University School of Medicine, Shanghai, 200025 China; 2 Department of Emergency, Ruijin Hospital, Shanghai Jiao Tong University School of Medicine, Shanghai, 200025 China

**Keywords:** Acute pancreatitis, Abdominal paracentesis drainage, Pelvic ascites

## Abstract

**Background:**

We sought to evaluate the effect of early short-term abdominal paracentesis drainage (APD) in moderately severe and severe acute pancreatitis (MSAP/SAP) with pelvic ascites.

**Methods:**

A total of 135 MSAP/SAP patients with early pelvic ascites were divided into the Short-term APD group (57 patients) and the Non-APD group (78 patients). The effects, complications, and prognosis of short-term APD patients were evaluated.

**Results:**

The baseline characteristics in the two groups were similar. The target days of intra-abdominal hypertension relief, half-dose enteral nutrition, duration of mechanical ventilation, length of intensive care unit stay (in days) and total hospitalization (also in days) were all lower in the Short-term APD group than in the Non-APD group (*P* = 0.002, 0.009, 0.004, 0.006 and 0.019), while the white blood cell count and serum C-reaction protein level decreased significantly more quickly (*P* < 0.01 and *P* < 0.05), and the prevalence of intra-abdominal infection was also significantly lower (*P* = 0.014) in the former than the latter. No complications occurred in early APD patients, and the microbial cultures of pelvic ascites were all negative. In addition, patients with early APD presented fewer cases of residual wall-off necrosis or fluid collection (*P* = 0.008) at discharge and had a lower incidence of rehospitalization and percutaneous catheter drainage and/or necrosectomy (*P* = 0.017 and 0.009).

**Conclusions:**

For MSAP/SAP patients with pelvic ascites, the early short-term APD is feasible and safe to perform, and it can decrease clinical symptoms, reduce intra-abdominal infection and shorten the hospital stay. It may also reduce the incidence of rehospitalization and surgical intervention.

## Background

The severity of acute pancreatitis (AP) has been defined by the revised Atlanta classification as mild AP (MAP), moderately severe AP (MSAP), and severe AP (SAP) [1]. Patients with MSAP/SAP almost always exhibit computed tomography (CT) findings of acute peripancreatic fluid collections [2]. Some early guidelines advocate conservative management of sterile fluid collections in AP [3, 4]. Walser et al. [5] demonstrated that the risk of infecting sterile collections through catheter drainage was 59% (13/22 patients) vs. 20% (3/15 patients) in collections that were only aspirated.

When Zerem et al. [6, 7] first reported that prolonged percutaneous catheter drainage (PCD) is more efficient for management of recurrent sterile fluid collections than is conservative treatment, especially in SAP patients with large and multiloculated fluid collections, their results was still challenged [8]. Other investigators from the same research team [9–12], reported on the use of abdominal paracentesis drainage (APD) of abdominal ascites ahead of PCD in AP patients. There are some differences with between APD and PCD: for example, the preference in APD is to enter the bilateral paracolic sulcus and lower abdomen to remove the ascites, and the correct time of APD is within four weeks of onset [9]. In contrast, PCD is usually undertaken in the region close to the pancreas to remove the infected peripancreatic fluid and the ideal time for intervention is usually 4 weeks after onset. Investigators have found that the APD for acute abdominal ascites is a protective factor for AP, with a dramatic decrease in intra-abdominal pressure (IAP), a low incidence of infection and alleviation of organ failure. In a recent report, Zerem et al. [13] accepted this opinion and considered the completion of APD ahead of PCD as a modification of the step-up approach in AP with fluid collections.

APD seems to be able to be implemented earlier in AP patients with low abdominal ascites, but its efficacy and safety still require clinical validation. In this study, we aimed to investigate whether early short-term APD is beneficial to MSAP/SAP patients with pelvic ascites.

## Methods

### Participant characteristics

We retrospectively analyzed the data of 267 patients with MSAP/SAP admitted to the Departments of Emergency and Critical Care Medicine of Ruijin Hospital, Shanghai Jiaotong University School of Medicine, Shanghai, China, from January 2015 to March 2019. The study was approved by the ethics committee of Ruijin Hospital, Shanghai, China (RJ2019-90). AP’s diagnostic criteria and severity classification were based on the revised Atlanta classification [1]. AP severity is classified as mild, moderately severe or severe. Organ failure was diagnosed using the modified Marshall scoring system [1]. Mild AP, the most common form, does not involve organ failure or local or systemic complications, and it usually resolves in the first week. MSAP involves the presence of transient organ failure, local complications or exacerbation of co-morbid disease(s). SAP involves persistent organ failure; that is, organ failure for > 48 h.

Study inclusion criteria were as follows: (1) age of ≥ 18 years but < 75 years; (2) the first onset of AP (3) admission within 72 h of onset; (4) meeting the diagnostic criteria of MSAP or SAP on admission; and (5) the presence of pelvic ascites (thickness ≥ 1 cm and volume ≥ 50 mL) under ultrasound. Conversely, the exclusion criteria were: (1) chronic pancreatitis; (2) pregnancy-related pancreatitis; (3) pancreatic cancer; (4) trauma or surgery-induced AP; (5) recurrent AP; and (6) patients with AP undergoing peri-pancreatic PCD, laparoscopic surgery or open surgery within 7 days of onset.

### Management of the early short-term APD

The localization of pelvic ascites and the puncture site were performed with ultrasonography. The applied drainage technique was the trocar method using a 16-gauge single-lumen central venous catheter (Baxter International Inc., Chicago, IL, USA). The catheter was introduced into the fluid collection via the most direct trans-peritoneal route, avoiding intervening bowel and solid organs. Once the catheter was in place, and the drainage was smooth, the fluid collection was drained passively.

Management of the catheter required the following steps: (1) disinfecting the puncture point every day and covering it with Tegaterm film (3 M Company, Saint Paul, MN, USA); (2) ensuring there was no lavage of the drainage tube; (3) allowing for pulling out and adjusting the depth of the drainage catheter, but do not inserting it again; and (4) pulling out the drainage catheter when the daily volume of drainage was < 100 mL for 2 consecutive days or the catheter indwelling time reached 7 days.

The daily volume and quality of drainage fluid were observed. The fluid samples obtained by the first puncture and collected every 48 h were sent for chemical examination and bacteriological culture. APD-related infection was defined as an intra-abdominal infection from 48 h after catheter indwelling to final extraction with evidence of a positive bacterial culture of ascites or catheter tip.

### Management of the disease and data collection

All patients in the trial received standard intensive care treatment, including fasting; gastrointestinal decompression; fluid resuscitation; oxygen therapy; mechanical ventilation (MV); nutritional supplements; and monitoring for respiratory, cardiovascular, and renal functions. Retrograde cholangiopancreatography (ERCP) with sphincterotomy and/or nasal biliary drainage was performed in patients with biliary pancreatitis.

During systemic observation, the relevant demographic data of patients included age, sex, body mass index (BMI), comorbidities and etiology. The classification of pancreatitis, severity classification, organ function evaluation, and definition of local complications are all referred to in the 2012 Atlanta International Consensus. Only early organ failures, including respiratory failure, circulatory failure and acute kidney injury (AKI), were included in the analysis. The APACHE II score and Ranson score were used to assess the clinical severity. The modified computed tomography severity index (MCTSI) was also used to assess the severity of pancreatic necrosis [14]. The amounts of regional effusion of the seven most common anatomical regions (I, lesser omental sac; II, the root of the mesenteric blood vessel; III, liver kidney recess; IV, splenorenal space; V, posterior space of right colon; VI, posterior space of left colon; VII, pelvic cavity) were evaluated by CT imaging. Every region that accumulated > 50 mL of effusion received one score. The IAP was determined by daily bladder pressure measurement. An IAP < 15 mmHg, daily maximum temperature < 37.5 °C and enteral nutrition (EN) ≥ 20 kCal/kg/day were considered as the parameters of clinical improvement, and the target days from onset of disease were recorded. Changes in inflammation markers, white blood cell (WBC) count and C-reaction protein (CRP) levels were recorded. Other indicators such as puncture complications, subsequent puncture and operation, hospital stay, and mortality were all evaluated. The information of six-months follow-up of patients discharge was also collected. All patient details were de-identified in the paper. The reporting of this study conforms to the Strengthening the Reporting of Observational Studies in Epidemiology guidelines [15].

### Statistical analysis

The statistical analysis was carried out using SPSS version 17.0 (IBM Corporation, Armonk, NY, USA). The quantitative data are tested for normality first. If they are in line with the normal distribution, they are expressed as the mean ± standard deviation values and compared using a *t*-test; if not, they are expressed as median values and compared with a non-parametric rank sum test. Categorical variables were expressed as numbers (%) and compared using the chi-square test. For small samples, Fisher’s exact test was used as appropriate. *P* < 0.05 was considered statistically significant.

## Results

### Patient grouping and general information

**Fig. 1:**
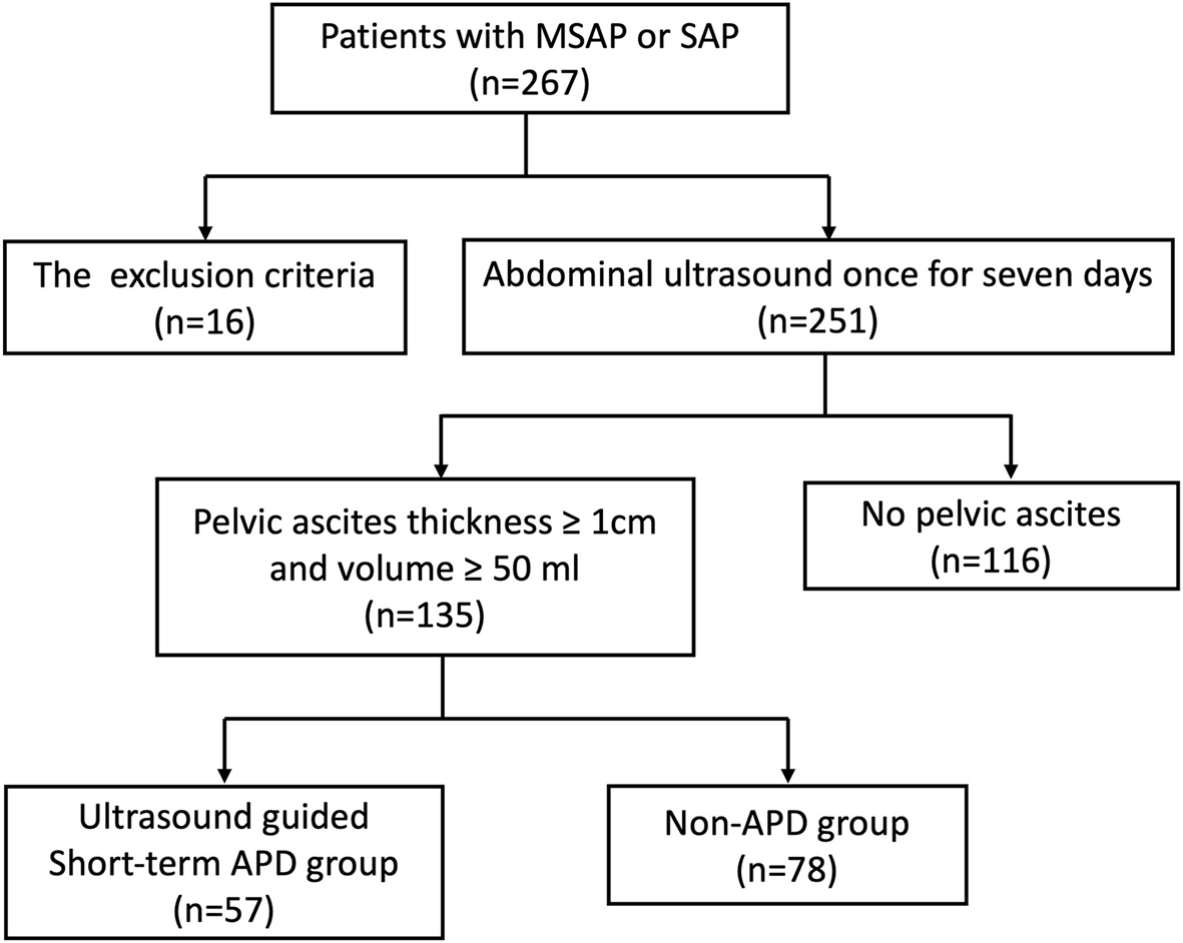
Flow diagram of moderately severe and severe acute pancreatitis patients

A total of 267 patients with MSAP/SAP were recruited (Fig. 1). Daily abdominal ultrasound was routinely performed to monitor pelvic ascites in every patient in the early stage (7 days after onset). Sixteen patients met the initial exclusion criteria. A total of 116 patients without pelvic ascites were not included and 135 patients with pelvic ascites were included in the analysis. Whether or not early ultrasound-guided APD for pelvic ascites should be implemented was dependent on the attending physician’s decision and the patient’s consent. Finally, 57 patients with pelvic ascites who underwent the APD were classified into the Short-term APD group, while 78 patients with pelvic ascites who did not undergo the APD were classified into the Non-APD group.

The patient’s general information, including age, sex, BMI, comorbidities, etiologies, APACHE II score, Ranson score, MCTSI value, regional fluid collection, IAP, early organ failure and serum biochemical test is compared in Table 1. There were no significant difference between two groups.

**Table 1:** Comparison of general information of patients

	Short-term APD (*n* = 57)	Non-APD (*n* = 78)	*P* value
**Age**	43.52 ± 15.63	46.12 ± 14.58	0.323
**Sex (male)**	31 (54.39%)	49 (62.82%)	0.325
**BMI**^**a**^	26.73 ± 4.75	25.52 ± 4.63	0.140
**Comorbidities**			
** Diabetes mellitus**	7	6	0.372
** Hypertension**	4	5	0.834
** Others**	2	4	0.978
**Etiology**			
** Biliary**	26 (45.61%)	34 (43.59%)	0.815
** Hypertriglyceridemia**	12 (21.05%)	15 (19.23%)	0.794
** Alcoholic**	8 (14.04%)	15 (19.23%)	0.575
** Idiopathic**	6 (10.53%)	8 (10.26%)	0.814
** Others**	5 (8.77%)	6 (7.69%)	0.927
**APACHE II**	13.47 ± 3.16	12.81 ± 3.40	0.253
**Ranson score**	5.01 ± 1.28	4.77 ± 1.23	0.273
**MCTSI**^**a**^	6.05 ± 1.72	5.91 ± 1.56	0.623
** Necrotic score**	3.27 ± 1.41	3.32 ± 1.38	0.837
**No. of regional fluid collection**	4.63 ± 1.12	4.57 ± 1.18	0.805
**IAP**^a^ **(mmHg)**	18.61 ± 3.47	18.09 ± 2.95	0.350
**Early organ failure**^**b**^	26 (45.61%)	38 (48.72%)	0.721
** Respiratory failure**	23 (40.35%)	31 (39.74%)	0.943
** Circulatory failure**	12 (21.05%)	23 (29.49%)	0.269
** AKI**^**a**^	17(29.82%)	15 (19.23%)	0.153
**Biochemical test**			
** Amylase (IU/L)**	601.60 ± 447.82	586.89 ± 406.32	0.843
** Hematocrit (%)**	42.37 ± 3.79	41.44 ± 4.00	0.175
** WBC**^a^ **count (×10**^**9**^**/L)**	14.75 ± 3.18	15.41 ± 3.62	0.273
** CRP (mg/L)**	67.97 ± 35.76	64.38 ± 33.20	0.549
** Ca**^**2+**^ **(mmol/L)**	2.02 ± 0.20	2.01 ± 0.20	0.775
** Creatinine (µmol/L)**	77 (39–542)	75 (35–437)	0.345

^a^*Abbreviations*: *BMI* Body mass index, *MCTSI* Modified computed tomography severity index, *IAP* Intra-abdominal pressure, *AKI* Acute kidney injury, *WBC* White blood cell, *CRP* C-reaction protein

^b^Early organ failure, i.e., organ failure in the early stage of AP

### APD of the pelvic ascites

**Fig. 2:**
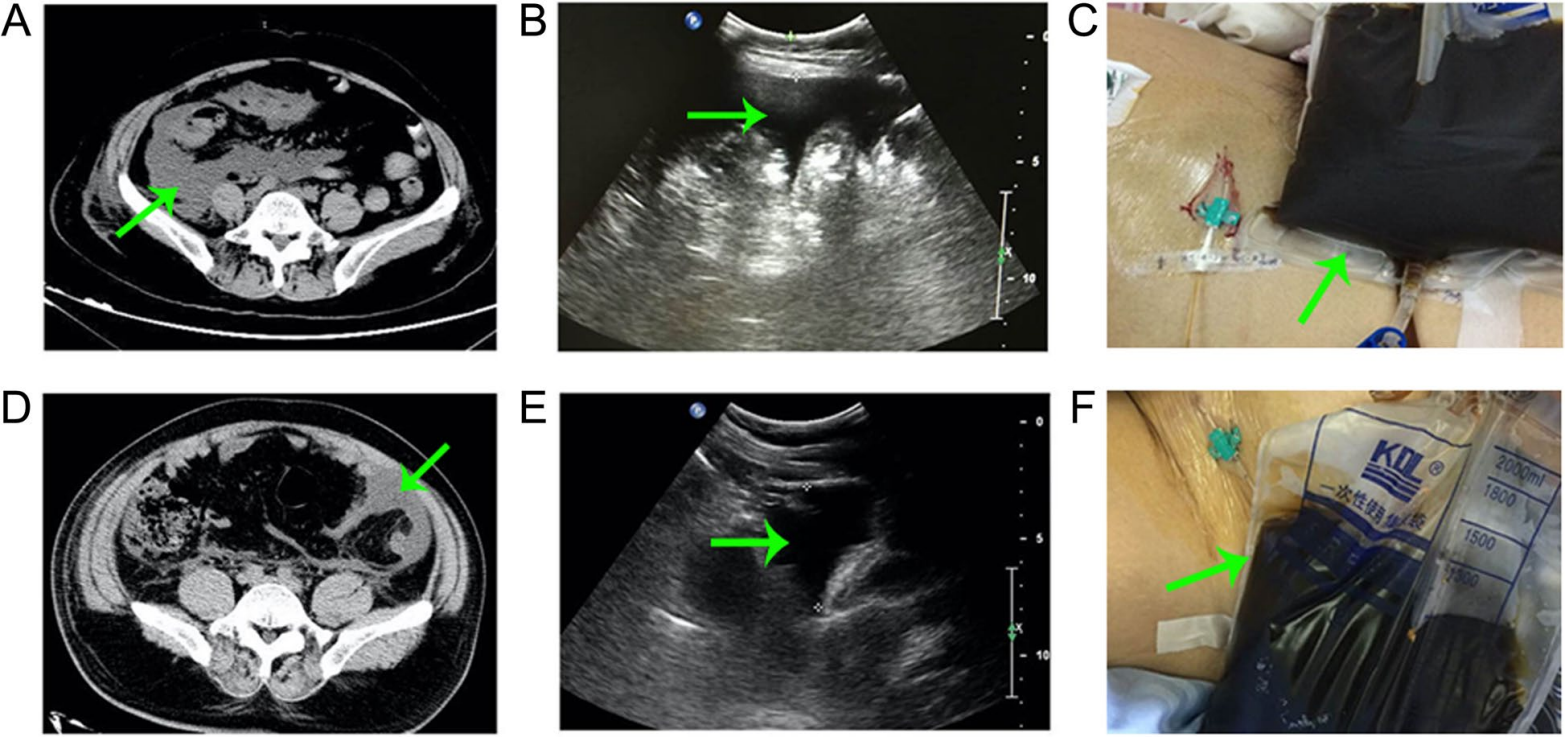
Pelvic ascites images obtained from two patients via computed tomography scan, ultrasound and macroscopy. **A**–**C** Computed tomography scan, ultrasound results and macroscopic pelvic ascites of one patient. **D**–**F** Computed tomography scan, ultrasound results and macroscopic pelvic ascites of another patient

In the Short-term APD group, the APDs were guided by ultrasound at 3.72 ± 1.13 days after the onset of AP. Forty-six patients underwent a single puncture with one drainage catheter, while 11 patients were implanted with two drainage catheters. No APD related complications occurred. The green arrows were the positions of the pelvic ascites in the CT images, ultrasound images and pelvic ascites images (Fig. 2A–F). The color of the pelvic ascites in 41 cases (71.93%) was dark brown (Fig. 2C, F). The median draining volume after APD was 1350 mL (between 400 mL and 3800 mL). The mean drainage duration time was 4.16 ± 1.64 days. The microbial cultures of pelvic ascites and catheter tips were all negative.

### Clinical effects and outcome

**Fig. 3:**
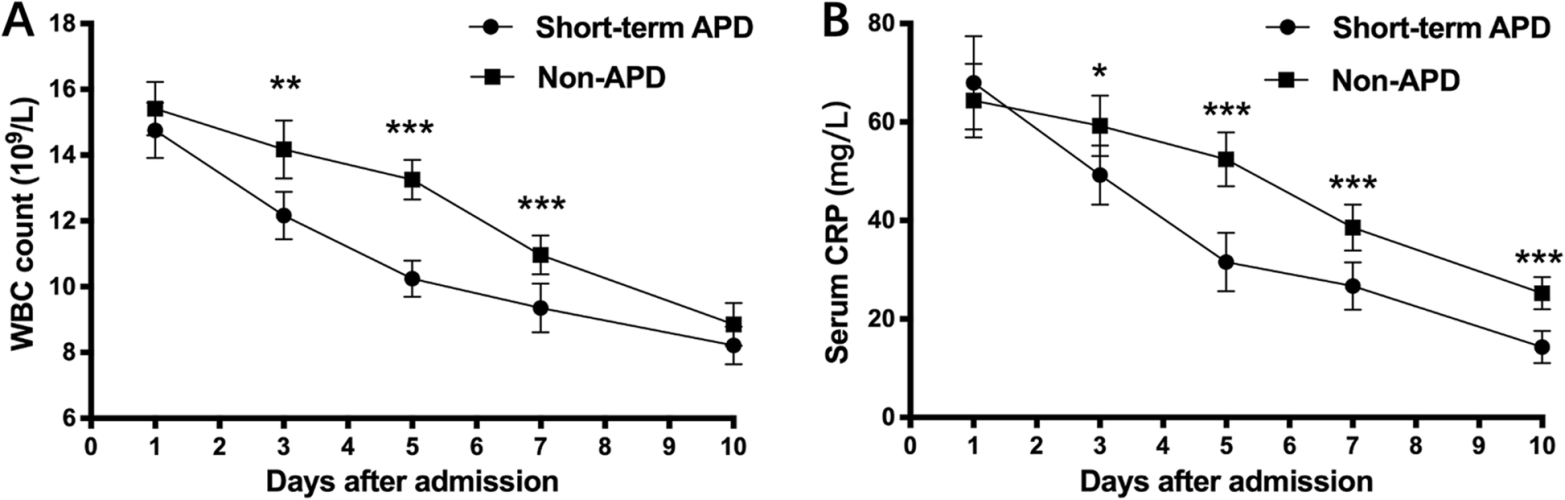
Comparison of WBC count and serum CRP level. **A** WBC count and **B** CRP level decreased more quickly in the Short-term APD group than in the Non-APD group. *p < 0.05, **p < 0.01 and ***p < 0.001

We also analyzed the clinical effects and outcomes of patients in both groups. As shown in Fig. 3, the WBC count decreased significantly more quickly in the Short-term APD group between 3 days and 7 days after admission than in the non-APD group (*p* < 0.01). Furthermore, the serum CRP level decreased significantly more quickly in the Short-term APD group between 3 days and 10 days after admission. (*p* < 0.05). When the clinical improvement was set to an IAP < 15 mmHg, daily maximum temperature < 37.5 °C and EN of ≥ 20 kCal/kg/day, the average target days of IAP and EN were significantly shorter in the Short-term APD group than in the Non-APD group (4.77 ± 2.81 days vs. 6.24 ± 2.52 days, *P* = 0.002; 6.53 ± 3.87 vs. 8.41 ± 4.25 days, *P* = 0.009) (Table 2).

**Table 2:** Clinical effects and outcomes

	Short-term APD (*n* = 57)	Non-APD (*n* = 78)	*P*-value
**Clinical improvement (days)**			
** IAP < 15 mmHg**	4.77 ± 2.81	6.24 ± 2.52	0.002
** Daily max temperature < 37.5 °C**	10.26 ± 7.28	11.44 ± 6.37	0.319
** EN**^**a**^ **≥ 20 kCal/kg/day**	6.53 ± 3.87	8.41 ± 4.25	0.009
**No. of early MV**^**b**^	19 (33.33%)	23 (29.49)	0.634
** Duration of MV**	7.68 ± 6.71	10.83 ± 5.82	0.004
**Intra-abdominal infection**	12 (21.05%)	32 (41.03%)	0.014
**Intervention in the late stage**			
** Later APD**^**b**^	5 (8.77%)	12 (15.38%)	0.378
** Peripancreatic PCD**^**a**^	16 (28.07%)	29 (37.18%)	0.267
** Open necrosectomy**	10 (17.54%)	25 (33.78%)	0.075
**Clinical outcome**			
** ICU stay**	20.75 ± 15.24	28.83 ± 19.75	0.006
** Total hospitalization days**	33.58 ± 19.75	41.75 ± 24.22	0.019
** Mortality**	6 (10.53%)	7 (9.00%)	0.137

^a^*Abbreviations*: *IAP* Intra-abdominal pressure, *EN* Enteral nutrition, *PCD* Percutaneous catheter drainage

^b^*Early MV* Mechanical ventilation in the early stage of AP, *later APD* Abdominal paracentesis drainage 7 days after disease onset

The incidence of early MV in the two groups was similar (33.33% vs. 29.49%, *P* = 0.634), while the duration of MV was significantly shorter in the Short-term APD group (7.68 ± 6.71 days vs. 10.83 ± 5.82 days, *P* = 0.004). There was no significant difference in circulatory failure and AKI incidence between the two groups (Table 2).

In the late stage, the prevalence of intra-abdominal infection was significantly lower in the Short-term APD group than in the Non-APD group (21.05% vs. 41.03%, *P* = 0.014). There were no significant differences between the two groups in the incidence of later APD, PCD, and open necrosectomy. Though the total mortality rates were similar between two groups (10.53% vs. 9.00%, *P* = 0.137), the lengths of ICU stay and total hospitalization were both significantly shorter in the Short-term APD group than in the Non-APD group (20.75 ± 15.24 vs. 28.83 ± 19.75 days, *P* = 0.006; 33.58 ± 19.75 vs. 41.75 ± 24.22 days, *P* = 0.019).

### Six-months follow-up of patients discharged

Survival patients without surgical necrosectomy were considered to have received successful conservative treatment. Meanwhile, unresolved necrosis and fluid collections were considered the main reasons for rehospitalization. We evaluated the necrotic score, presence of wall-off necrosis (WON), and fluid collections of the discharged patients (Table 3). Then the patients were followed up until six months of discharge, focusing on rehospitalization, PCD, and surgical necrosectomy.

**Table 3:** Six-month follow-up after patient discharge

	Short-term APD (*n* = 45)	Non-APD (*n* = 50)	*P* value
**On discharge**			
** MCTSI**^**a**^ **-necrotic score**	3.56 ± 1.40	3.45 ± 1.44	0.658
** No. of WON**^**a**^ **or fluid collection (> 100 mL)**	2.20 ± 1.11	2.78 ± 0.99	0.008
**Follow-up after 6 months**			
** Rehospitalization**	11 (24.40%)	24 (48.00%)	0.017
** Intra-abdominal infection**	9 (20.00%)	20 (40.00%)	0.059
** PCD**^**a**^ **and/or necrosectomy**	10 (22.22%)	24 (48.00%)	0.009

^a^*Abbreviations*: *MCTSI* Modified computed tomography severity index, *WON* Wall-off necrosis, *PCD* Percutaneous catheter drainage

We found that 45 of 57 (78.95%) patients in the Short-term APD group and 50 of 78 (64.10%) patients in the Non-APD group survived and were discharged as successfully conservatively treated patients (Table 3). Although there was no significant difference in necrotic pancreatic score between the two groups, the number of WON or fluid collection (> 100 mL) was significantly less in the Short-term APD group than in the Non-APD group (2.20 ± 1.11 vs. 2.78 ± 0.99, *p* = 0.008). During 6 months of follow-up after discharge, the incidence rates of rehospitalization and PCD and/or necrosectomy in the Short-term APD group were both lower than those in the Non-APD group (24.40% vs. 48.00%, *P* = 0.017; 22.22% vs. 48.00%, *P* = 0.009). The main reason for rehospitalization in both groups was a secondary intra-abdominal infection.

## Discussion

The removal of peritoneal ascitic fluid using APD is possibly advantageous in a subset of patients with severe acute pancreatitis [16]. APD leads to significantly decreased trends in all-cause mortality, length of stay and expenses when compared to the conventional ‘step-up’ treatment [17–20]. However, these results must be interpreted with caution because of the lack of high-quality evidence. Here, we once again verified the feasibility and effectiveness of early short-term APD in severe acute pancreatitis, and we documented some improvements to the APD procedure.

In previous studies, the mean time of APD was about 11 days after onset. Gou et al. [21] reported their experience of early PCD 7 days after onset in a letter, and the point-in-time is consistent with ours. Our APD’s mean time was 3.72 days, which is earlier than the 7 days that Gou et al. reported. Liu et al. [9] suggested the right paracolic sulci, left paracolic sulci, and the pelvic cavity for APD among the most common anatomical regions of the liquid collection. Our collected region is pelvic ascites. In fact, it is difficult to collect the liquid in the paracolic sulci. This liquid can flow into the pelvic cavity and form a thick collection which is easy and safe for drainage with bedside ultrasound. Our drainage catheter was a thinner catheter (16 g) that differs from those used by others (8 to 22 F pigtail catheters). Such a thin catheter could drain pelvic ascites with a median drainage volume of 1350 mL. We considered that a thin catheter could reduce catheter-related infections and muscle injury.

Under the above APD process, no complications occurred in this study. In Liu et al.’s report [11], eight (6.3%) cases of APD-related complications occurred among 126 patients, including four puncture site hemorrhages, two abdominal hemorrhages, one colon perforation, and one catheter occlusion. Fewer data on these initial complications could be found in other reports. Some reports documented secondary intra-abdominal infections in APD patients, including APD-related infections and infections due to other causes, with rates ranging from 31.7 to 83.3%,. Experience has been shown that catheter change, enlargement or lavage, and delayed withdrawal increase the risk of infections. The concept of “no-touching and off in time” of catheter management in this study could be suggested in clinical practice.

The clinical effects of early APD were also verified in this study, which included releasing IAP, shortening the MV time, decreasing the WBC count and serum CRP, realizing an earlier half-dose EN, reducing later infections, and finally shortening the ICU stay and total hospitalization stay. The incidence rates of later APD, PCD, necrosectomy and mortality in the Short-term APD group were decreased during the hospitalization, but the data had no statistical significance. We also considered the long-term prognosis of successfully conservative patients, especially those with unabsorbed necrosis and fluid collection, which will easily undergo infection and rehospitalization. Interestingly, the incidence of rehospitalization, intra-abdominal infection, PCD, and/or necrosectomy in early APD patients was lower 6 months after discharge.

Similar effects were also reported in recent studies. Liu et al. [9] indicated that APD was beneficial to patients by reducing inflammatory factors, postponing further interventions, and delaying or avoiding multiple organ failure. Liu et al. [11] showed that APD did not increase infectious complications and infection-related mortality. Li et al. [19] reported the improvements in WBC count, serum amylase, CRP and serum calcium after early drainage. Gou et al. [21] showed the role of early drainage of ascites in decreasing IAP, serum high-sensitivity-CRP and inflammatory cytokines. Liang et al. [22] found that APD could improve the tolerance of EN in AP. Zerem et al. [6] indicated that the APD ahead of PCD is safe and beneficial for patients as it reduces inflammatory factors, postpones further interventions, and delays or avoids multiple organ failure. In addition, the positive effect of APD on the prognosis has also been reported. Lu et al. [18] reported a systemic review of APD with a result of reduced all-cause mortality. Formanchuk et al. [23] indicated that using catheter drainage methods could reduce mortality and improve treatment outcomes in AP patients complicated by fluid collections. As an initial minimal invasive intervention for MSAP/SAP patients, its direct effect is to drain pelvic ascites rich in trypsin, inflammatory media, and toxic substances. Meanwhile, the intra-abdominal hypertension can be relieved. In clinical observation, we noticed that APD not only effectively drains the pelvic ascites but also reduces the effusion in bilateral paracolonic sulcus and splenorenal space, which might be the effect of fluid flow under intra-abdominal hypertension, albeit without an effect on the pancreatic necrosis. Thus, APD combined with PCD and necrosectomy may improve the prognosis of necrotizing pancreatitis.

In conclusion, the early short-term APD of pelvic ascites is feasible and safe for MAP/SAP patients since it can effectively relieve early intra-abdominal hypertension, shorten the time of MV, realize half-dose EN earlier, and reduce intra-abdominal infections. In addition, early APD patients have a lower incidence of rehospitalization and necrosectomy among successfully conservative patients. Thus, we contend that the early APD could be an initial step in managing MSAP/SAP with pelvic ascites.

However, our study had some limitations. This was a retrospective study with few samples, and large-scale studies are needed to support our findings. The APD was applied to a subgroup of SAP patients. About 50% of patients with MSAP/SAP presented with pelvic ascites in this study. The implementation of APD also depends on ultrasound, puncture, and nursing technologies.

## Data Availability

The datasets used and/or analyzed during the current study are available from the corresponding author on reasonable request.
